# Eco-friendly approach for the effective leaching of valuable metals (Ni, Co, Mn) from spent lithium-ion batteries employing natural reductants

**DOI:** 10.1039/d6ra03283d

**Published:** 2026-06-01

**Authors:** Wajiha Khalid, Muhammad Kaleem Khosa, Sadaf Fatima, Awal Noor, Sadaf Qayyum, Mohd Farhan

**Affiliations:** a Department of Chemistry, Government College University Faisalabad Pakistan mkhosapk@yahoo.com; b Department of Chemistry, College of Science, King Faisal University Al-Ahsa Saudi Arabia anoor@kfu.edu.sa

## Abstract

The widespread utilization of lithium-ion batteries (LIBs) in energy storage systems has led to an accumulation of discarded LIBs. Recycling has been implemented as a novel approach to alleviate the detrimental impacts of battery waste and facilitate the recovery of precious metals. This innovative method utilizes an eco-friendly hydrometallurgical process for metal leaching, employing biodegradable mixed organic acids, specifically citric acid (C.A.) and tartaric acid (T.A.), alongside sugarcane bagasse, an agricultural byproduct. These experiments indicate that around 84% of Ni, 88% of Co, 95% of Li, and 93% of Mn were extracted under optimal circumstances of 1.5 : 1.5 mol L^−1^ mixed acids (C.A. : T.A.) concentration, 0.6 g g^−1^ bagasse dose, 15 g L^−1^ slurry density, 50 °C temperature, 50 minutes leaching duration, and 400 rpm agitation speed. Moreover, experimental findings validated that organic compounds in bagasse improved the leaching efficiencies of precious metals. The leaching kinetics governed by chemical reactions are accurately represented by an Avrami model, which provides apparent activation energies of 47 kJ mol^−1^ for Co, 44 kJ mol^−1^ for Li, 46 kJ mol^−1^ for Mn, and 45 kJ mol^−1^ for Ni. This process turns agricultural waste into a valuable asset, reducing the environmental impact of discarded lithium-ion batteries.

## Abstract 2

Citric and tartaric acids as lixiviant and sugarcane bagasse as green reductant, have been successfully applied to leach valuable metals from spent lithium-ion batteries.

## Graphical abstract

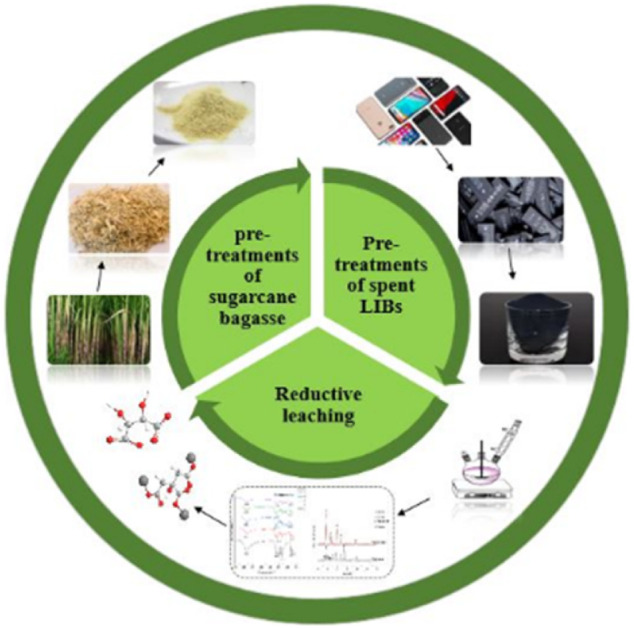

## Introduction

The lithium-ion battery is a revolutionary technology which drives many reliable systems.^1^ Since their invention by Sony in 1990, LIBs have emerged as a significant achievement in modern battery electrochemistry.^2^ LIBs have gained dominance in the current global rechargeable battery market due to their long shelf life, lightweight design, high energy density, and temperature resilience. As an electrochemical power source, LIBs are used in a wide range of applications, including portable electronics, military, navigation, medical equipment, and electric vehicles. Due to widespread adoption, the LIB industry has witnessed extensive growth.^3^ The limited lifespan of LIBS (1–3 years) due to high operating temperatures, technical cycling complications, and rate performance has led to increased accumulation of spent LIBs.^4^ According to estimates, by 2030, about 11 million metric tons of spent LIBs must be disposed of, of which only 5% will be recycled.^5^ Thus, 95% of untreated spent LIBs will cause severe environmental, economic, and health impacts. Therefore, sustainable strategies are required to separate LIB components into various fractions and re-feed them into production.^6–9^ The entire recycling process comprises two fundamental categories: physical and chemical processes. The hydrometallurgical process is well developed for recycling spent LIBs.^10^ The advantages of this process include high selectivity, reduced acid consumption, lower gas emissions, and improved recycling efficiency as compared to the conventional methods (*e.g.*: pyrometallurgy, direct recycling and biometallurgy) as shown in Table 1. A typical hydrometallurgical process consists of pretreatments followed by leaching and selective precipitation of metals.^11^ Several inorganic acids are effective for high metal recovery, but their use has adverse effects, including energy consumption, emissions of gases (Cl_2_, SO_3_, and NO_*x*_), and excessive water consumption.^12^ To overcome these challenges, eco-friendlier organic acids, such as citric acid,^13^ lactic acid,^14^ oxalic acid,^15^ ascorbic acid,^16^ and tartaric acid^17^ were employed. The effectiveness of the hydrometallurgical process for recycling metals depends on the stability of metal ions in aqueous solution. In spent LIBs, metals (Mn, Co, Ni) are primarily in insoluble trivalent and tetravalent form.^18^ The introduction of a reducing environment enhanced the leaching efficiencies of metals. Inorganic compounds such as sulphur dioxide, iron and sodium sulphite are effective reductants^19^ but their safety hazards and instability in acid solution limit their sustainability. In addition to that, H_2_O_2_ has been extensively employed due to its strong green reducing ability and clean decomposition behaviour. However, despite these advantages, it remains commercially synthesized chemical reductant that enhance chemical dependency and reagent cost. Therefore, in recent studies, bio-based organic reductants such as grape seeds,^15^ tea waste,^20^ glucose,^21^ orange peel waste,^22^ and litchi peel powder^23^ have attracted a considerable attention as sustainable and cost-effective alternatives. Comparison table of natural reductants has been provided in Table 2.

**Table 1:** Comparison between recycling technologies

Recycling processes	Process steps	Current scale of application	Recycling cost/time	Advantages	Disadvantages
Direct process^24^	Size reduction	Laboratory	Low/fast	Low emission of greenhouse gases and low energy demand	Low process robustness and limited material purity
Pyrometallurgy^25^	Combustion	Industrial	High/fast	Application flexibility and simple pretreatments	High emission of greenhouse gases and high energy demand
Biometallurgy^26^	Bioleaching	Laboratory	Low/slow	Low emission of greenhouse gases and low energy demand	Long processing time and low process robustness
Hydrometallurgy^27^	Leaching	Industrial	Moderate/moderate	Application flexibility and low energy demand	Equipment maintenance/corrosion and requires effluents post-treatment

**Table 2:** Comparison of bio-based reductants for leaching efficiencies of metals

Reductant	Conditions	Recovery	Metals	Leaching agent used
Orange peel^22^	Temp. (100 °C), citric acid concentration (1.5 M), reaction duration (4 h), and slurry density (25 g mL^−1^)	80–99%	Li, Ni, Co, Mn	Citric acid
Glucose^28^	Temp. (95 °C), H_2_SO_4_ concentration (3 M), reaction duration (2 h), and slurry density (25 g L^−1^)	More than 95%	Li, Co	Sulphuric acid
Grape seed^15^	Temp. (80 °C), C_4_H_6_O_5_ concentration (1.5 M), reaction duration (3 h), and slurry density (20 g L^−1^)	More than 90%	Li, Co	Malic acid
Waste areca powder^29^	Temp. (89 °C), H_2_SO_4_ concentration (3 M), reaction duration (2 h), and L/S ratio of 20 : 1	About 99.9%	Li, Ni, Co, Mn	Sulphuric acid
Bell pepper^30^	Temp. (70 °C), H_2_SO_4_ concentration (2 M), reaction duration (2 h), and slurry density (20 g L^−1^)	More than 90%	Li, Ni, Co, Mn	Sulphuric acid

Sugarcane bagasse is a by-product of sugar production that is rich in cellulose, hemicellulose, and lignin. It is an agro-waste left after the sugarcane juice is extracted. Its good reactivity, low cost and availability makes it a better reductant than traditional ones.^31^ It was used as a reducing agent for Mn leaching from polymetallic materials, such as iron-rich Mn dioxide ore, and suggested a novel utilization of bagasse as a green reductant for the extraction of metals from spent LIBs.

In this study, a mixture of environmentally degradable citric and tartaric acids, together with sugarcane bagasse, was used to extract valuable metals using hydrometallurgical principles. Herein, ultrasonic-assisted separation of cathode material was adopted as a green alternative to conventional chemical pretreatments. The innovative aspect of our current work lies in using agricultural waste as a versatile green reductant and lixiviant for spent LIB recycling. This eco-driven approach demonstrates the waste + waste = resource strategy, which promotes the circular economy, cost-effectiveness, and sustainability. It is intended that this innovative process will emerge as a promising alternative for reclaiming metals from spent LIBs.

## Experimental

### Materials and reagents

Spent LIBs from different mobile phones and bagasse were collected from the local market, with a mass fraction of 20 wt% Mn, 28 wt% Co, 9 wt% Li and 6 wt% Ni. The composition was determined by ICP-OES analysis. Analytical-grade chemical reagents were used in this experiment, including citric acid, tartaric acid, ethanol, and NaCl.

### Sample pre-treatments

Collected batteries were first discharged in a 10 wt% NaCl solution for 24 h to prevent the risk of explosion and fire. When the batteries' voltage was less than 2 V (measured with a voltmeter), they were dismantled into cathode sheets, anode sheets, separators, plastics, and steel casings. To separate the cathode material from Al-foil, the cathode sheets were immersed in 100 mL ethanol and subjected to ultrasonic cleaning (3 h). The cathode was then filtered and heated at 700 °C in a muffle furnace for 3 h to burn off organics, such as the binder (PVDF).^32^ Sugarcane bagasse collected from the local market was washed with distilled water to remove dust and other impurities. After cleaning, it was dried and ground into a fine powder to increase its surface area. Powdered bagasse was subjected to alkaline treatment (1% NaOH) at 100 °C for 1 h to break down the lignin.

### Reductive leaching

All leaching reactions were conducted in a 250 mL glass beaker equipped with a magnetic stirrer. The powder of cathode material was weighed according to its solid-to-liquid ratio (10–25 g L^−1^) and then added to a mixture with varying quantities of citric acid (0.5–2.5 mol L^−1^) and tartaric acid (0.5–2.5 mol L^−1^). After heating at 30–70 °C, the bagasse (0.3–0.7 g per 1 g of cathode powder) was added to the solution as a reductant. The reaction mixture was agitated at 200–600 rpm for 80 min. The pH of the solution before and after leaching was 2 and 2.5, respectively. At scheduled intervals, a series of liquid samples (1.0 mL) were collected from the mixture using syringe filters (0.2 µm) to get the leaching efficiencies of each metal for kinetic analysis. After leaching, the undissolved black residue was separated by filtration.

### Analytical methods

ICP-OES (Teledyne Leeman Labs Prodigy 7) analysis was used to determine the concentrations of valuable metals, including Ni, Co, Mn, and Li, in the leachate. The morphology of the cathode powder and the leaching residue was identified by SEM (Nova NanoSEM 450). For phase identification of the sample residue, powder X-ray diffraction (D8 Advance, Bruker) with Cu Kα radiation was used. FT-IR spectroscopy (Bruker, Billerica, MA, USA) was used to identify changes in functional groups in leachate and bagasse during the experiment, with a spectral range from 400–4000 cm^−1^. The leachate spectrum was obtained using a UV-visible spectrophotometer (UH5300-HITECH).

### Leaching mechanism

As biodegradable, cost-effective leaching agents, both citric and tartaric acids participate, to leach valuable metals from spent LIBs. Being a triprotic acid, citric acid exhibits comparatively stronger chelation ability because on dissolution it releases hydrogen ions, which are responsible for the stabilization of dissolved metal species in solution. Due to the dissociation of 1 M citric acid, about 3 M H^+^ ions are produced as shown in eqn (1)–(3).^33^

1 H_3_Cit → H_2_Cit^−^ + H^+^, *K*_a1_ = 7.4 × 10^−4^

2 H_2_Cit^−^ → HCit^2−^ + H^+^, *K*_a2_ = 1.7×10^−5^

3 HCit^2−^ → HCit^3−^ + H^+^, *K*_a3_ = 4.0 × 10^−7^

Tartaric acids also participate to metal complexation by providing additional protons that support metal dissolution. As a diprotic and dicarboxylic acids, it dissociates as given in eqn (4) and (5).^34^

4 H_2_C_4_H_4_O_6_ → HC_4_H_4_O^−^_6_ + H^+^, p*K*_a1_ = 2.72

5 HC_4_H_4_O^−^_6_ → C_4_H_4_O^2−^_6_ + H^+^, p*K*_a2_ = 4.79

**Fig. 1:**
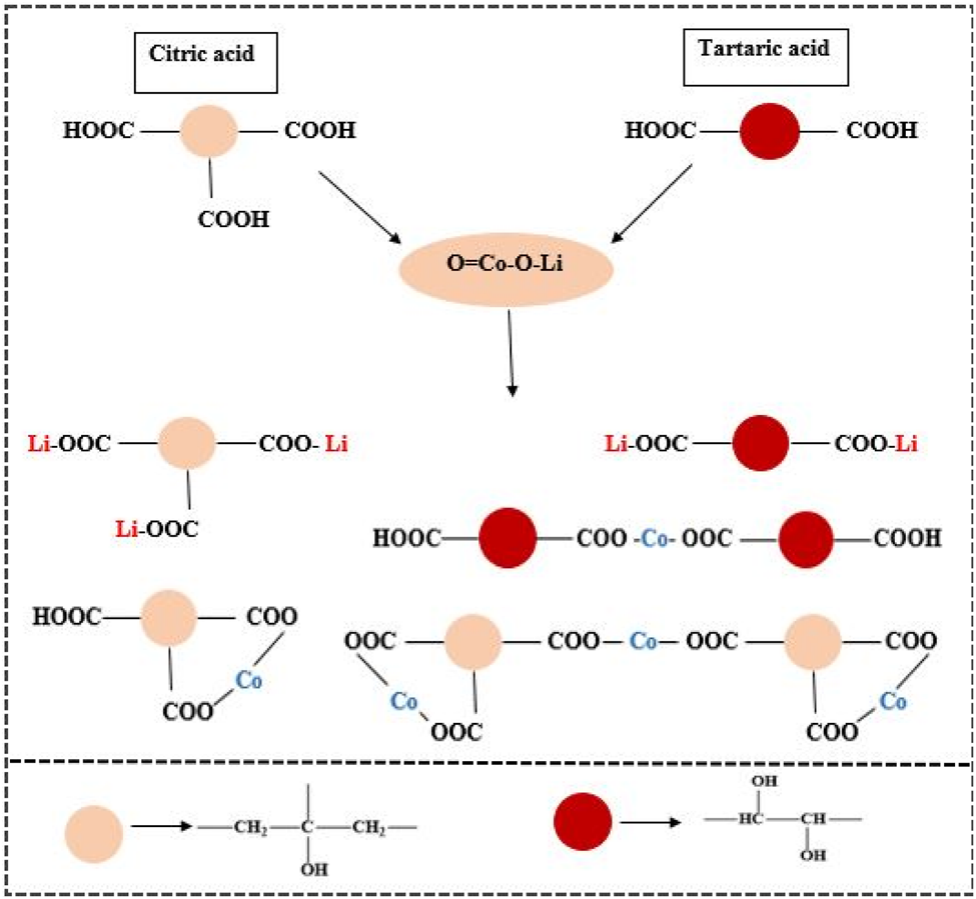
Possible metal complexes of mixed organic acids.

Therefore, the combined acid system (citric and tartaric acid) may increase leaching efficiencies by complementary acidification and complexation effects, as shown in Fig. 1.

**Fig. 2:**
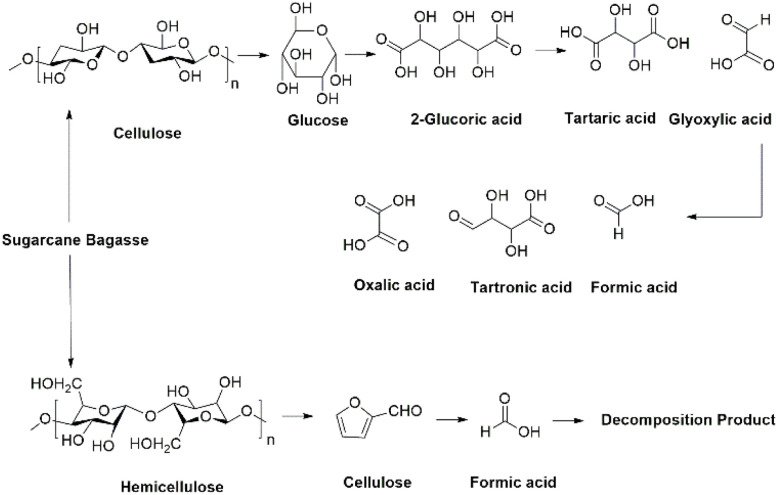
Possible degradation pathway of sugarcane bagasse.

During the dissolution process, bagasse acts as a reductant. Under acidic conditions, the cellulose and hemicellulose components of sugarcane bagasse are hydrolyzed into low-molecular-weight reducing sugars, along with degradable products as shown in Fig. 2.^28^ These reducing sugars contain reducing groups which convert insoluble metal ions into their solubilize forms, which are essential for enhancing the overall leaching efficiencies of metals. The synergistic effect of mixed acids and sugarcane bagasse enhances metal dissolution in aqueous media, thereby achieving higher leaching efficiencies.

### Optimization parameters

#### Acids concentration, slurry density, reductants dosage, temperature and time

**Fig. 3:**
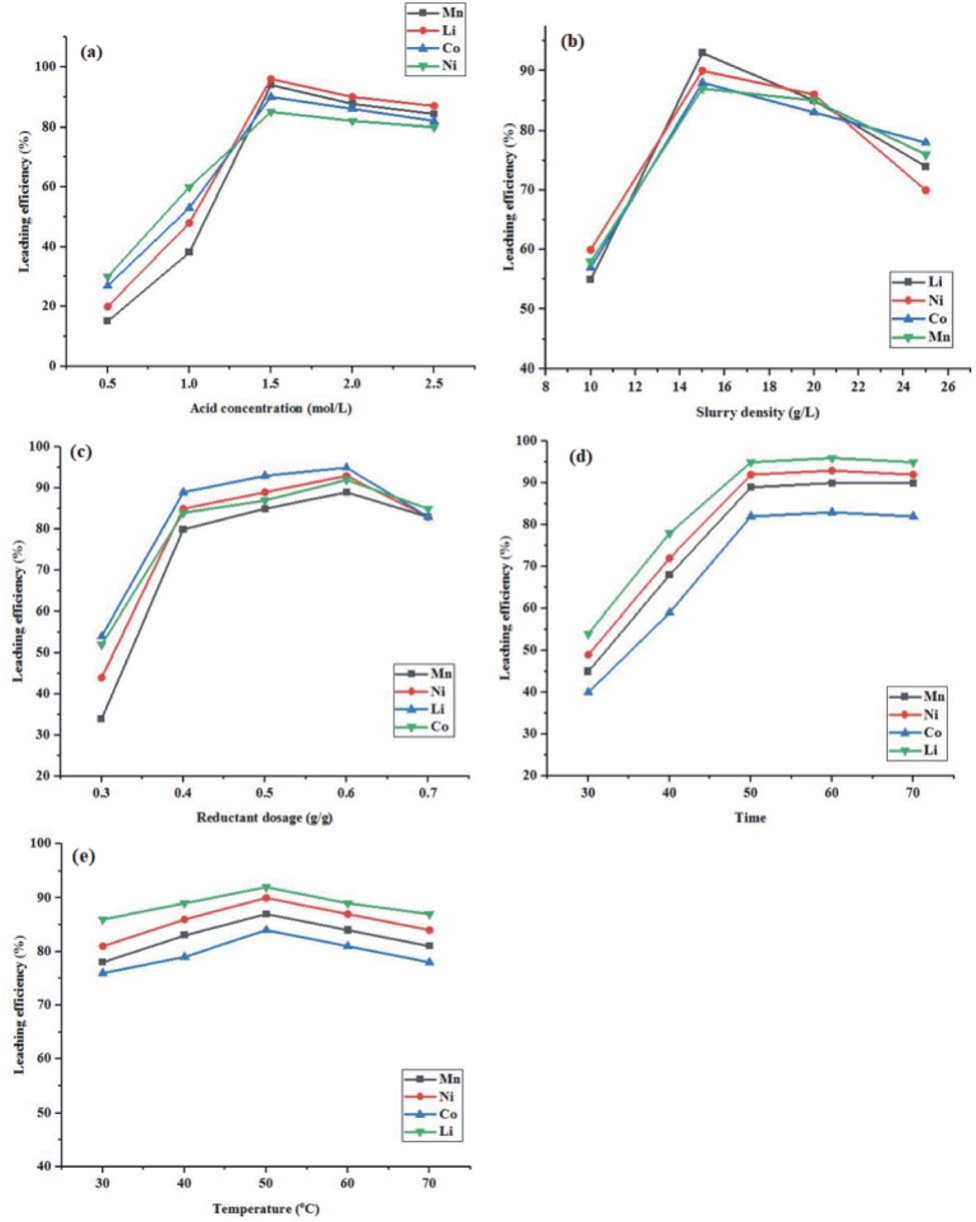
(a) Influence of acid concentration; (b) slurry density; (c) reductants dosage; (d) time; (e) temperature.

The efficiency of the leaching process is highly dependent on the concentration of the mixed acids. Fig. 3(a) shows how the concentration of mixed acids affects the leaching of metals. All other parameters, including the slurry density of 20 g L^−1^, temperature of 50 °C, and time of 50 minutes, were held constant throughout the leaching process. Lower metal leaching efficiencies were observed at lower concentrations during mixed-acid leaching, according to the experimental results. With the high availability of H^+^ ions, which promote dissolution, the leaching efficiencies of Li, Ni, Mn, and Co peak at 95%, 84%, 93%, and 88%, respectively, at a concentration of 1.5 M. However, as the concentration continues to rise, 0.5 M increase from 1.5 to 2 M is numerically small but it can still elevate the concentration of acids leading to saturation and ultimately this restricts the ion diffusion away from the particle surface. At 2 M and 3 M, the higher availability of leaching agents promotes the formation of stable metal citrate or tartrate precipitates. These precipitates can form a passive layer on spent battery particles, further hindering leaching efficiency. Therefore, 1.5 M acid is the ideal concentration for effective metal leaching. Fig. 3(b) shows the relationship between slurry density and metal leaching efficiency, with all other parameters held constant: 1.5 M : 1.5 M mixed acids (C.A. : T.A.) concentration, 0.4 g g^−1^ bagasse dosage, 80 minutes of time, and 40 °C temperature. The effect of pulp density (5–25 g L^−1^) on metal leaching efficiency was studied under optimal conditions. The results show that leaching efficiency increased with pulp density up to 15 g L^−1^. The slightly lower efficiency at 10 g L^−1^ may be associated with reduced solid concentration, leading to lower reaction frequency between solid particles and reactive species. Similar trends have been reported in hydrometallurgical leaching systems, where an intermediate pulp density is often optimal due to a balance between lixiviant and mass transfer limitations.^35–37^^.^ The highest leaching efficiencies were obtained at 15 g L^−1^ for cobalt (86%), nickel (87%), manganese (85%), and lithium (93%).

Further, increase in slurry density to 20 g L^−1^ and 25 g L^−1^ decrease metal extraction, due to reduced lixiviant availability per unit mass of solid and increased diffusion limitations at higher solid loadings. As a result, 15 g L^−1^ was selected as the optimal pulp density for subsequent experiments.

Fig. 3(c) shows how the amount of reductant has a significant effect on the solubility of metals. The ideal parameters for studying this effect are a concentration of 1.5 M : 1.5 M mixed acids (C.A. : T.A.), a slurry density of 15 g L^−1^, an 80 minute timer, and a temperature of 40 °C. The leaching efficiencies of Li, Ni, Mn, and Co increase to 93%, 88%, and 87%, respectively, in bagasse-assisted mixed-acid leaching when the bagasse dose is varied from 0.2 to 0.6 g g^−1^. This is because the sugars in bagasse have a reducing effect, which accelerates the reaction by converting trivalent or tetravalent metals into divalent forms that are more soluble. Lower leaching efficiencies were observed with a dose increase of 0.7 g g^−1^. An excess bagasse dosage adsorbs metal ions and lowers their leaching efficiency.^38^ Thus, the ideal reductant dosage is 0.6 g g^−1^. As demonstrated in Fig. 3(d), the leaching efficiency of Co, Ni, Mn, and Li is affected by the duration of leaching. This effect is studied in an ideal setting with a 1.5 M : 1.5 M mixed acid concentration, a 0.6 g g^−1^ bagasse dosage, a 15 g L^−1^ slurry density, and a leaching temperature of 40 °C. Co, Li, Ni, and Mn had leaching efficiencies of 40%, 53%, 57%, and 45%, respectively, following 30 minutes of reaction. As the leaching reaction was allowed to continue for an additional 50 minutes, the leaching efficiency increased to 78%, 93%, 90%, and 86%, respectively. Leaching efficiencies are unaffected by longer durations.^14^ Therefore, 50 minutes is the sweet spot for leaching length. Leaching efficiencies of Ni, Co, Mn, and Li at temperatures ranging from 30 to 70 °C are shown in Fig. 3(e). Experimental settings included a 1.5 M : 1.5 M mixed acid concentration, a dosage of 0.6 g g^−1^ bagasse, a slurry density of 15 g L^−1^, and a leaching time of 50 minutes to study the effect of temperature on metal leaching efficiency. At a lower temperature of 30 °C, the experimental results showed that around 85% of Li, 82% of Ni, 75% of Co, and 76% of Mn leached. These findings proved that metal leaching is not an appropriate process at low temperatures. Leaching efficiencies of Li, Ni, Co, and Mn increased to 90%, 88%, 82%, and 84%, respectively, at 50 °C, demonstrating that temperature is a crucial factor influencing metal leaching.^15^ However, as the temperature rises to 70 °C, metal leaching decreases because acid degradation occurs at elevated temperatures. For metal leaching, 50 °C is the ideal temperature.

### Kinetic analysis

To gain a better understanding of the leaching process, kinetic analysis was carried out under optimal conditions of 0.6 g g^−1^ bagasse dosage, 15 g L^−1^ slurry density, 1.5 M : 1.5 M mixed-acid concentration, and 50 °C, at various time intervals. The resulting data were utilized to compare the fitting efficiencies of different models (Shrinking core model with chemical control and diffusion-controlled models, eqn (6) and (7), and the empirical model, eqn (8)) to assess metal leaching kinetics.

6 1−(1 − *x*)^1/3^ = *k*_1_*t*

7 1 − 3(1 − *x*)^2/3^ + 2(1 − *x*) = *k*_2_*t*

8 {−ln(1 − *x*)}^2^ = *K*_emp_*t*

**Fig. 4:**
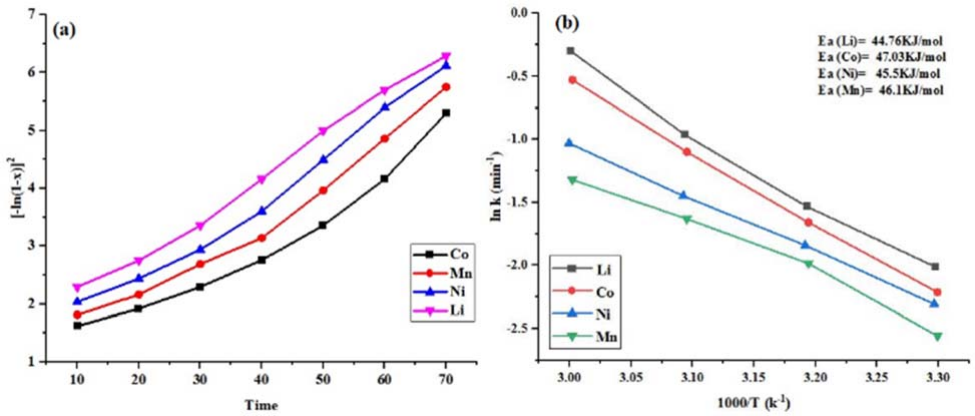
(a) Kinetic analysis of {−ln(1 − *x*)}^2^*vs.* t at 50 °C leaching temperatures for Li, Co, Ni, Mn and (b) Arrhenius plot for the leaching of valuable metals at various temperatures.

Compared with other models (as shown in Table 3), the Avrami equation exhibited a superior fit (*R*^2^ > 0.95), showing that it is a best fit for the experimental leaching data. Furthermore, Avrami exponent (*n*) smaller than 1 indicating that the kinetic behaviour is predominantly controlled by the heterogeneous reaction mechanism involving diffusion-related effects. This suggests that the reaction rate gradually decrease with the passage of time.^39^ that's why the Avrami equation is employed to analyze multi-metal leaching for some solid–liquid heterogeneous reactions. The *K*_emp_ values for all the metals have been measured at each temperature using the slope of the linear fit to the plots of {−ln(1 − *x*)}^2^ against *t* (as shown in Fig. 4(a)). The *K*_emp_ value was further used to determine the apparent activation energies of the metals by applying the empirical Arrhenius equation (eqn (9)).

9 *K* = *A*e^−*E*_a_/*RT*^

Here, *R* = universal gas constant (J K^−1^ mol^−1^), *K* = reaction rate constant (min^−1^), *E*_a_ = apparent activation energy (kJ mol^−1^), *A* = Arrhenius constant, *T* = absolute temperature (K).

**Table 3:** Comparative table of different kinetic models

Temp. (°C)	Shrinking core model (surface reaction)			Shrinking core model (diffusion controlled)			Avrami model		
	ln *k*	*R* ^2^	*n*	ln *k*	*R* ^2^	*n*	ln *k*	*R* ^2^	*n*
30	−0.0254	0.9268	0.0951	−0.0249	0.717	0.0963	−0.0351	0.923	0.0967
40	0.0611	0.918	0.0993	0.0647	0.7714	0.1003	0.0441	0.9318	0.1036
50	−0.0384	0.9319	0.1162	−0.0386	0.8422	0.1306	−0.0526	0.9527	0.1191

Fig. 4(b) shows the Arrhenius plot between ln *k* and reciprocal temperature (1/*T*) to measure the apparent activation energy values of metals. The respective linear slope of this plot is denoted as –*E*_a_/*R*, and the intercept as ln *A*. The estimated activation energies for Co, Ni, Mn, and Li were 47 kJ mol^−1^, 45 kJ mol^−1^, 46 kJ mol^−1^, and 44 kJ mol^−1^, respectively. The lower activation energy of Li indicated rapid leaching, as its valence state remained unchanged during the reaction. Cobalt's valence state in the cathode material was Co(iii), which got reduced to soluble Co(ii); therefore, its activation energy is greater. The calculated activation energies of all metals were greater than 40 kJ mol^−1^, indicating a chemically controlled process.

## Results and discussion

### XRD analysis

**Fig. 5:**
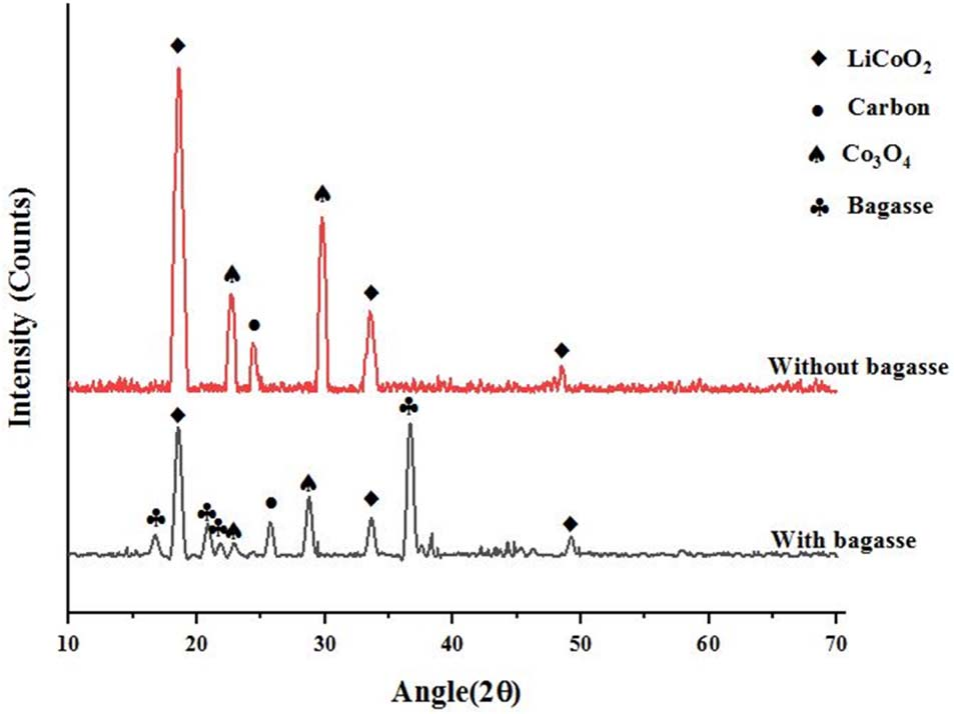
XRD diffractogram.

Fig. 5 demonstrates the XRD pattern of the leaching residue obtained using mixed acid leachate. This spectrum was collected over a range of 10° to 70° (2*θ*). The high-intensity peaks at 18.59° and 33.50°, and a weak diffraction peak at 48.48°, indicate the presence of LiCoO_2_, which remains insoluble during leaching. In comparison, the presence of a sharp peak at 24.42° represents the carbon residue that cannot be leached during the dissolution reaction.^40^ The peaks at 22.68° and 29.77° are characteristic of Co_3_O_4_. It also represents the XRD spectra of the sample residue obtained *via* bagasse-assisted mixed-acid leaching. The sharp peaks at 18.47°, 49.22°, and 33.57°, due to LiCoO_2_, were less intense because the crystal structure of the cathode material deforms, reducing the metal to a divalent, solubilized form. Some new peaks were introduced due to the incorporation of bagasse. The sharp peaks at 16.80°, 36.66°, 20.87°, and 21.80° were due to the crystallization intensity of cellulose and hemicellulose components of bagasse. No further peaks of crystalline material were detected, indicating that, under optimal leaching conditions, the components were water-soluble.

### UV-visible spectroscopy

**Fig. 6:**
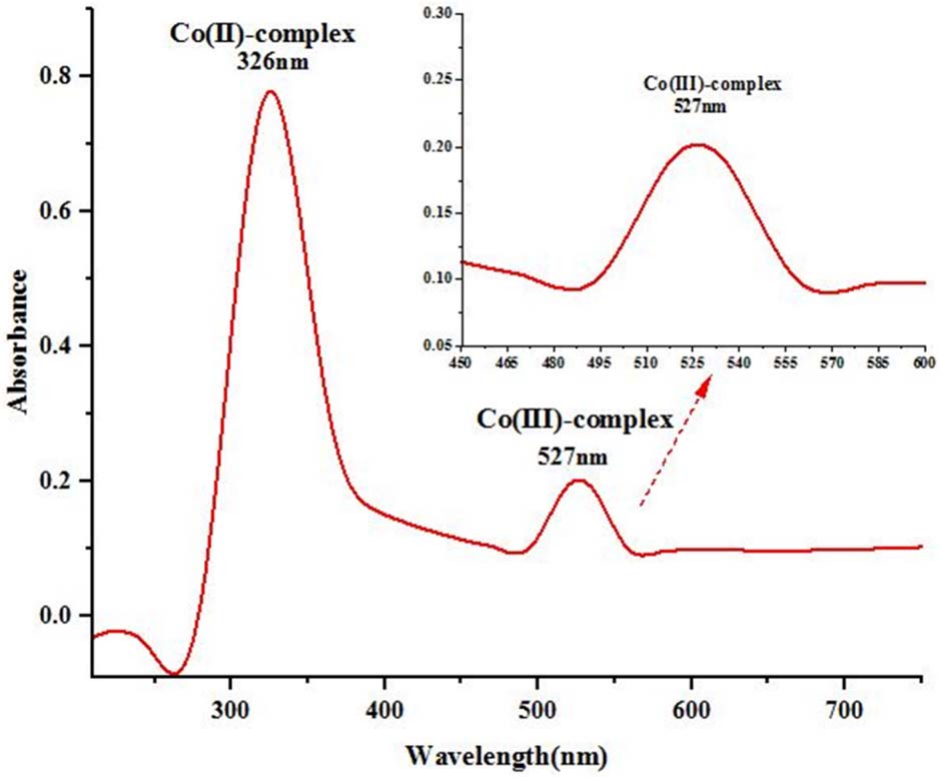
UV-visible spectra of leachate.

UV-visible spectra of bagasse-assisted mixed acid leachate are shown in Fig. 6. The maximum absorption (*λ*_max_) peaks at 326 nm and 527 nm are attributed to M(ii)-L and M(iii)-L, respectively (M = Co; L = citrate, or tartrate).^41,42^ The strong Co(ii) peak indicated that the introduction of bagasse into the reaction mixture enhanced metal leaching by facilitating the dissolution of metal oxides.

**Fig. 7:**
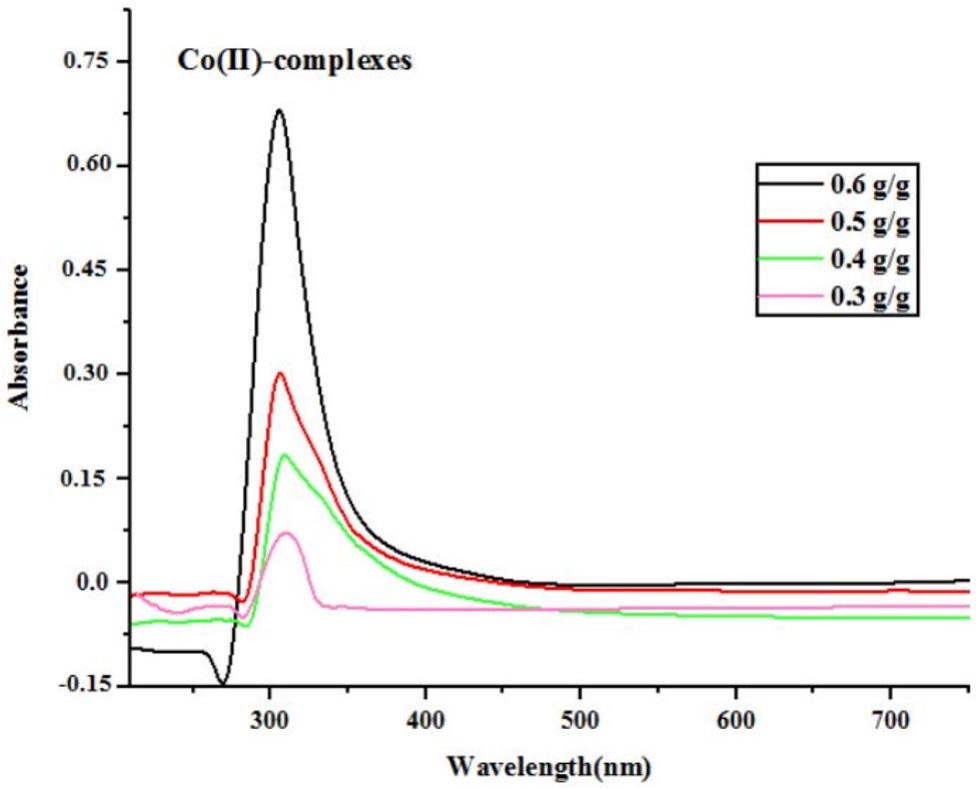
UV-visible spectra of leachate at various reductant dosages.

Moreover, the effect of various bagasse dosages on leaching efficiencies of Co-complexes was confirmed by UV-vis characterization. From UV-visible spectra, it can be seen that absorption peak at 326 nm was due to Co(ii)-complex (Fig. 7). As the reductant dosage rise from 0.3 g g^−1^ to 0.6 g g^−1^, the absorption intensity significantly rise from 0.1 to 0.7, the increase in absorbance at 326 nm with increasing bagasse dosage (0.3–0.6 g g^−1^) indicates higher concentration of Co(ii) related species in solutions, suggesting enhanced redox activity and leaching efficiency, this trend suggests a possible shift in cobalt speciation from Co(iii) to Co(ii) with increasing sugarcane bagasse.

### FT-IR analysis

**Fig. 8:**
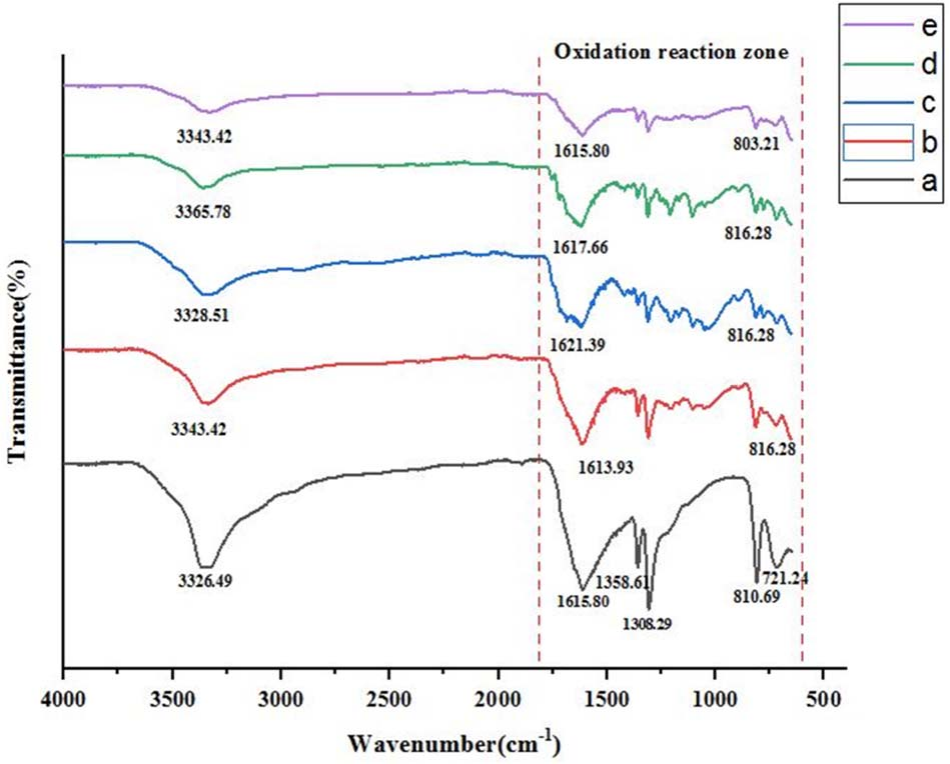
FT-IR spectra of (a) leaching residue using mixed acid leachate, (b) leaching residue using mixed acid and bagasse, and (c, d, e) at different optimal conditions.

Fig. 8 illustrates the FT-IR spectra of the leaching residue obtained using mild organic acids, with and without bagasse. It shows that the characteristic peaks at 3326.64, 1615.80, 1308.29, and 810.69 cm^−1^ are due to O–H stretching, C=O stretching, C–H bending, and M–O bond vibrations. These functional groups confirm the presence of undissolved carboxylic acids and cathode powder in the leaching residue. It represents the FT-IR spectrum of the leaching residue obtained after incorporating sugarcane bagasse under optimized conditions. The characteristic peaks appeared at 3343.42, 1613.93, 1312.05, and 816.28 cm^−1^, with additional peaks due to the introduction of bagasse into the reaction. These new peaks at 1425.70, 1207.65 and 1041.79 cm^−1^ are due to aromatic C=C, C–O stretches, and C–O–C stretches.^26^ The decline in O–H peak intensity may indicate changes in hydroxyl-containing functional groups during the leaching process. The decrease in C=C and C=O peak intensities, suggests possible interactions between organic ligands and metal species, which may facilitate complexation during leaching.

The reduction of M–O peak intensity may be associated with the dissolution or transformation of metal oxides phases and changes in coordination environment during leaching. However, FT-IR analysis alone does not provide direct evidence of metal valence changes and is therefore considered supportive rather than definitive.

### SEM analysis

**Fig. 9:**
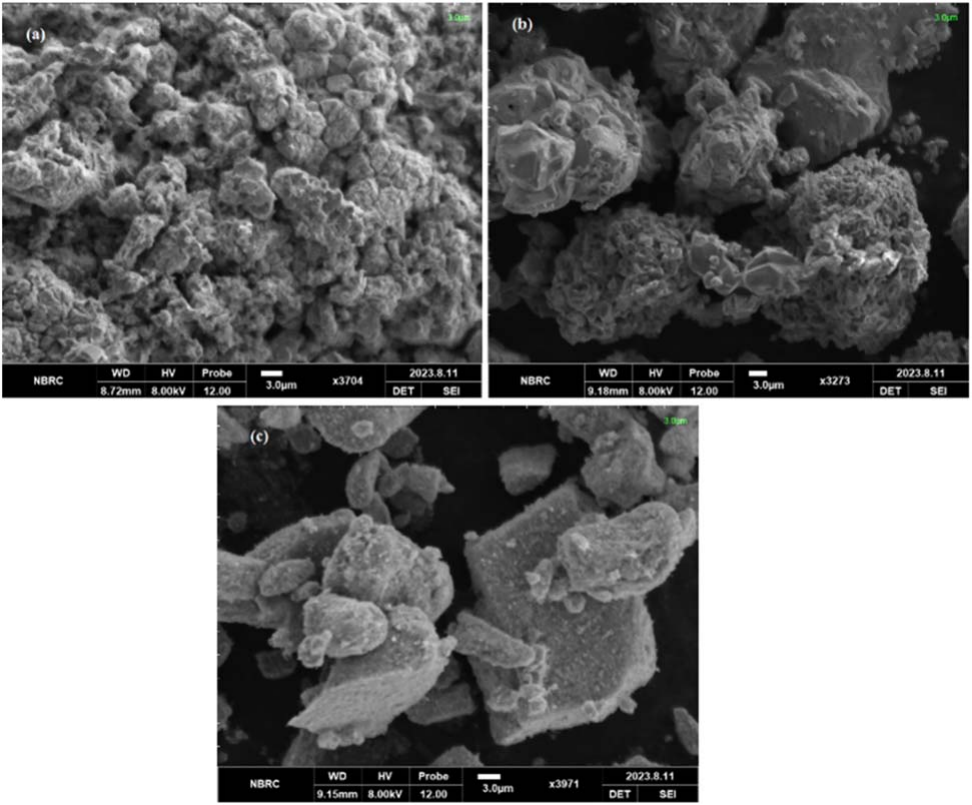
SEM images of (a) cathode powder after calcination, (b) leaching residue using mixed acid leachate, and (c) sample residue obtained using mixed acid and bagasse leaching.

Fig. 9(a) shows a low-magnification SEM image of the calcined cathode material. The micrograph shows agglomerated particles with fused regions. Large secondary particle clusters are visible in the structure. Particle fusion and extensive agglomeration are clearly observed in the calcined cathode powder, resulting in dense secondary particle clusters.

Fig. 9(b) illustrates the SEM images of the sample residue obtained after mixed acid leaching. The surface exhibits a porous morphology with visible voids. The particles show irregular shapes and non-uniform size distribution. A rough surface texture is observed across the residue. Discontinuous and uneven surface features are present in the microstructure.

The SEM image of the residue after leaching with mixed acids and bagasse is presented in Fig. 9(c). The image contains irregularly shaped particles with visible surface pores. Surface voids and fragmented regions are clearly observed. Moreover, the large particles are embedded within the voids of the porous matrix. The surface shows heterogeneous morphology with distinct void spaces.

A clear contrast is observed between the calcined cathode powder and leaching residues across Fig. 9(a–c) indicating a transition from agglomerated structures to increasingly porous and heterogeneous surface morphologies.

## Conclusions

The study aimed to analyze the potential of LIBs recycling using sugarcane bagasse in mixed acid media. Herein, citric and tartaric acids are used as lixiviants, and sugarcane bagasse is used as a green reductant. The influence of different leaching conditions, such as acid concentration, bagasse dosage, slurry density, temperature, time, and agitation speed, was analyzed. This reductive complexing mechanism demonstrates that about 84% of Ni, 88% of Co, 95% of Li and 93% of Mn were leached under optimal conditions of 1.5 M : 1.5 M mixed acids (C.A. : T.A.) concentration, 0.6 g g^−1^ bagasse dosage, 15 g L^−1^ slurry density, 50 °C temperature, 50 min leaching time at 400 rpm agitation speed. Experimental results confirmed that addition of bagasse enhanced the dissolution of valuable metals (Ni, Mn, Co and Li) due to the oxidative breakdown of reducing sugars present inside bagasse that reduce the insoluble Ni(iii), Mn(iv) and Co(iii) complexes into soluble Ni(ii), Mn(ii) and Co(ii) complexes. The apparent activation energies of Li (44 kJ mol^−1^), Co (47 kJ mol^−1^), Ni (45 kJ mol^−1^) and Mn (46 kJ mol^−1^) showed that the reaction was chemically controlled. UV-vis analysis of the leachate showed a reduction of Co(iii)-complex to Co(ii)-complex upon the introduction of bagasse. This process is an efficient strategy for recovering valuable metals from spent LIBs, featuring lower environmental impact, cost-effectiveness, and reduced acid consumption.

## Author contributions

Muhammad Kaleem Khosa: conceptualization, supervision, project administration, writing—review and editing. Wajiha Khalid: methodology, formal analysis, investigation, writing—original draft preparation. Sadaf Fatima: investigation, writing—original draft preparation. Awal Noor: writing—review and editing, funding acquisition. Sadaf Qayyum: writing—review and editing, funding acquisition. Mohd Farhan: writing—review and editing, funding acquisition.

## Conflicts of interest

There are no conflicts to declare.

## Data Availability

The original contributions presented in the study are included in the article, further inquiries can be directed to the corresponding authors.
